# Deacetylation of Transcription Factors in Carcinogenesis

**DOI:** 10.3390/ijms222111810

**Published:** 2021-10-30

**Authors:** Marta Halasa, Kamila Adamczuk, Grzegorz Adamczuk, Syeda Afshan, Andrzej Stepulak, Marek Cybulski, Anna Wawruszak

**Affiliations:** 1 Chair and Department of Biochemistry and Molecular Biology, Medical University of Lublin, Witolda Chodźki 1 St., 20-093 Lublin, Poland; martahalasa@umlub.pl (M.H.); kamilaadamczuk@umlub.pl (K.A.); andrzejstepulak@umlub.pl (A.S.); marekcybulski@umlub.pl (M.C.); 2 Independent Medical Biology Unit, Medical University of Lublin, Kazimierza Jaczewskiego 8b St., 20-090 Lublin, Poland; grzegorzadamczuk@umlub.pl; 3 Institute of Biomedicine and FICAN West Cancer Centre, University of Turku and Turku University Hospital, 20520 Turku, Finland; syeda.afshan@utu.fi

**Keywords:** HDAC, histone deacetylase inhibitors (HDIs), transcription factors

## Abstract

Reversible Nε-lysine acetylation/deacetylation is one of the most common post-translational modifications (PTM) of histones and non-histone proteins that is regulated by histone acetyltransferases (HATs) and histone deacetylases (HDACs). This epigenetic process is highly involved in carcinogenesis, affecting histone and non-histone proteins’ properties and their biological functions. Some of the transcription factors, including tumor suppressors and oncoproteins, undergo this modification altering different cell signaling pathways. HDACs deacetylate their targets, which leads to either the upregulation or downregulation of proteins involved in the regulation of cell cycle and apoptosis, ultimately influencing tumor growth, invasion, and drug resistance. Therefore, epigenetic modifications are of great clinical importance and may constitute a new therapeutic target in cancer treatment. This review is aimed to present the significance of HDACs in carcinogenesis through their influence on functions of transcription factors, and therefore regulation of different signaling pathways, cancer progression, and metastasis.

## 1. Introduction

Cancer is a leading cause of premature death next to cardiovascular diseases, with over 19 million new patients and 9.9 million fatalities worldwide in 2020. The latest statistics clearly show that cancer incidence and mortality have increased significantly, partly due to socioeconomic factors, including aging and population growth, as well as factors related to people’s behavior and living habitat [1,2].

Cancer is defined as a disorganized cells state, where cells undergo uncontrolled division assaulting host tissue and other tissues (metastasis), annexing critical cell survival resources at the expense of healthy cells, and ultimately causing cell death [3]. These events occur due to progressive series of genetic aberrations and mutations of oncogenes and tumor suppressor genes. Genetic mutations are caused due to inherited and environmental factors and switch the normal cells toward precancerous cells, multiplying and finally evolving into cancer cells [4]. In addition to genetic changes, epigenetic changes are critical in carcinogenesis as they could cooperate with genetic abbreviations that deliver cancer phenotypes. Epigenetics could explain heritable changes in gene expression, which do not follow DNA sequence alterations. Carcinogenesis depends on both genetic and epigenetic alterations, but unlike genetic changes, epigenetic alterations are reversible. Epigenetic mechanisms include modifications on histone proteins, DNA methylation, and regulation of gene expression by non-coding RNAs and microRNAs. All these mechanisms are critical for tumor initiation, progression, and metastasis [5,6], and they have been considered innovative biomarkers or new targets in targeted therapy in various types of cancers [7,8,9,10]. Histone proteins undergo reversible acetylation by opposite working enzymes: histone acetyltransferases (HATs) and histone deacetylases (HDACs). Histone acetylation by HATs is critical for providing sufficient space for local transcription events, making chromatin active, whereas histones deacetylation by HDACs leads to chromatin deactivation [11].

**Figure 1:**
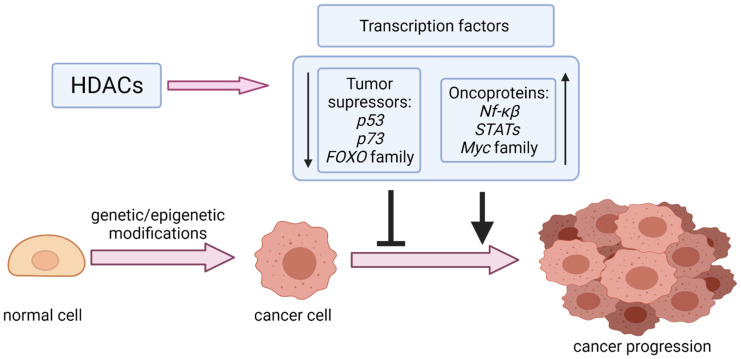
Modulation of transcription factors (tumor suppressors and oncoproteins) by HDACs in cancer progression.

In addition to histones, non-histone proteins also undergo reversible acetylation by HATs and HDACs. HDACs are critical post-translational modifiers with distinct roles in human carcinogenesis, giving a different biological effect depending on the type of tumor. They can be categorized into two groups. The first group consists of Zn^2+^ dependent HDACs, divided into four classes depending on their homology, sequence similarity, and expression patterns. Class I and IIa are comprised of four members (HDAC 1, 2, 3, 8, and HDAC4,5,7,9, respectively), class IIb possesses two members (HDAC6 and 10), and class IV only one (HDAC 11). The second group, referred to as the sirtuin family, consists of 7 members (from SIRT1 to SIRT7), which require nicotinamide adenine dinucleotide (NAD) for their activity [10]. HDACs overexpression has been confirmed in various cancers [12,13], providing evidence for the importance of their activity in cancer progression. HDACs activity is essential for controlling gene expression by deacetylation of critical for tumor suppression and tumor development transcription factors such as tumor suppressor p53 (TP53, best known as p53) [14,15], forkhead box (FOX) proteins [16], nuclear factor kappa-light-chain-enhancer of activated B cells (NF-κB) [17], and Myc-family proteins [18] (Figure 1). HDACs also affect signaling mediators, including phosphatase and tensin homolog (PTEN), signal transducer and activator of transcription 3 (STAT3) [19,20], protein kinase B (Akt) [21], and β-catenin [22], as well as other nuclear proteins like Ku70 [23] and structural proteins such as α-tubulin [24].

The role of tumor suppressors (TSs) should be emphasized, as they act as cellular protectors that prevent the development of tumors by inhibiting proliferation and inducing apoptosis of cancer cells through their involvement in signaling pathways and regulation of proteins responsible for cancer initiation, progression and invasion [25]. Reversibly, oncoproteins directly induce the occurrence of the neoplastic phenotype due to the perturbation of signal transduction, cell growth, and differentiation [26]. Among TSs and oncoproteins, transcription factors (TFs) constitute a large group of proteins modulated by post-transcriptional modifications. TFs modulate gene expression by binding to the promoter regions of their target genes (or enhancers’ regions) and thus modulate gene transcription [27]. Both steps are known to be modulated by epigenetic modifications of the specific TFs, in majority phosphorylation and acetylation [28,29,30]. In general, post-translational modifications of TFs significantly impact chromatin remodeling, cell cycle regulation, apoptosis, and autophagy.

Notably, the HDAC activity is modulated by activators and inhibitors (HDIs). The latter have shown their potential against hematological malignancies, although they were not efficient enough against solid tumors [31]. So far, 4 HDIs have been approved by The Food and Drug Administration (FDA): vorinostat (SAHA) in cutaneous T-cell lymphoma (CTLC) treatment, belinostat, and romidepsin in peripheral T-cell lymphoma (PTCL) treatment, and panobinostat in multiple myeloma treatment [32]. However, these inhibitors are used in cancer therapy; they are not selective against specific HDAC isoforms, resulting in serve side effects [33]. Nowadays, many other HDIs are tested in preclinical trials, and some of them seem to be promising anti-cancer tools. However, each HDIs can uniquely affect different cancer cells giving contrary outcomes in terms of cancer types [34].

In this review, we present the mechanism of HDACs action against selected tumor-related transcription factors highly expressed in cancer progression mentioning the role of HDIs in these events.

## 2. Tumor Suppressors

### 2.1. p53 Deacetylation by HDACs

p53 tumor suppressor encoded by the *TP53* gene becomes activated under stress conditions and during uncontrolled cell division. Activated p53 regulates a huge amount of genes involved in various biological processes due to its transcriptional properties. P53 induces cell cycle arrest by p21 activation to stop uncontrolled cell growth, and it triggers pro-apoptotic genes such as *BAX* to cause cell death when the DNA damage is beyond repair [35].

This section presents the importance of modulation of p53 by HDACs and HDIs, including biological consequences. HDIs reduce p53 expression at mRNA and protein levels [36,37,38] and increase p53 acetylation enabling its activation and stabilization [37,38]; therefore, HDIs are of great importance in search of targeted therapy.

#### 2.1.1. p53 Is Deacetylated and Destabilized by SIRT1

**Figure 2:**
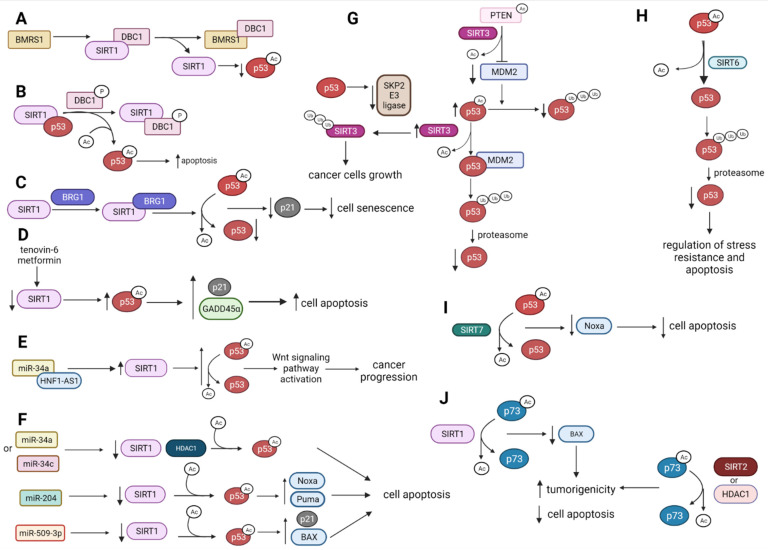
The biological effects of the p53-Ac and p73-Ac deacetylation catalyzed by SIRTs and HDACs. ↑—upregulation, ↓—downregulation (**A**) BMRS1 disrupts the interaction between SIRT1 and DBC1, decreasing the p53-Ac level. (**B**) Phosphorylated DBC1 binds to SIRT1 that dissociates the SIRT1–p53 complex and simulates p53 acetylation leading to apoptosis. (**C**) BRG1 binds to SIRT1, which results in increased p53-Ac deacetylation and inhibits p53/p21-mediated cell senescence. (**D**) Downregulation of SIRT1 expression by specific inhibitors is associated with the upregulation of p53-Ac, which leads to the upregulation of proteins involved in apoptosis. (**E**) Repression of miR-34a/SIRT1/p53 feedback loop by HNF1A-AS1 leads to a decrease in p53-Ac and activation of the Wtn signaling pathway, which, in turn, enhances cancer progression. (**F**) SIRT1 is regulated by several miRNAs, leading to a change of the p53-Ac level and modulation of apoptosis. (**G**) The influence of SIRT3 on p53Ac deacetylation and p53 degradation by the proteasome. (**H**) SIRT6 deacetylates p53-Ac, which is ubiquitinated and degraded by the proteasome. (**I**) SIRT7 deacetylates p53-Ac, which leads to downregulation of Noxa and ultimately the inhibition of cell apoptosis. (**J**) SIRT1, SIRT2, and HDAC1 can deacetylate p73-Ac, leading to a decrease in apoptosis and increased cell proliferation and tumorigenicity.BMRS1—breast cancer metastasis suppressor 1; DBC1—breast cancer 1; BRG1—brahma-related gene-1; HNF1A-AS1—HNF1A-antisense 1 RNA1.

The control of p53 protein stability is essential to maintain its tumor suppressor functions. The p53 stability can be modulated by deleted in breast cancer 1 (DBC1) protein, which acts as an endogenic inhibitor of SIRT1 [39]. DBC1 interplays with SIRT1, reducing its deacetylase activity and maintaining the stable acetylated p53 (p53-Ac) level. DBC1 interacts with breast cancer metastasis suppressor 1 (BMRS1), interrupting DBC1-SIRT1 association and giving a conclusion that BRMS1 can downregulate SIRT1-mediated p53 deacetylation upon DNA damage (Figure 2A) [40]. DBC1 becomes phosphorylated by ataxia telangiectasia-mutated (ATM), ataxia telangiectasia, and Rad3-related (ATR) kinases in response to DNA damage. Phosphorylated DBC1 (DBC1-P) binds to SIRT1, leading to the dissociation of the SIRT1–p53 complex and the promotion of p53 activation via its acetylation and p53-dependent apoptosis [41] (Figure 2B).

In colorectal cancer (CRC) cells, suppression of nicotinamide phosphoribosyl transferase (NAMPT) decreases SIRT1 activity, which results in the upregulation of p53 acetylation. Subsequently, p53-Ac promotes G0/G1 cell arrest by inducing p21 expression and enhancing caspase-3-mediated apoptosis [42]. The activity of SIRT1 deacetylase can be enhanced by brahma-related gene-1 (BRG1) protein, a subunit of the SWI/SNF chromatin-remodeling complex. BRG1 binds to SIRT1 and increases SIRT1-mediated deacetylation of p53, leading to its destabilization. Knockdown of BRG1 promotes cell senescence and inhibits CRC growth by modulating the SIRT1/p53/p21 signaling axis (Figure 2C) [43]. 

SIRT1-dependent p53 deacetylation can be inhibited by HDIs, which has significant clinical importance. Preclinical studies focused on p53 deacetylation by SIRT1 showed that some HDIs can be considered as potential anti-cancer treatment tools. Tenovin-6, an SIRT1 inhibitor, in combination with metformin, an activator of AMP-activated protein kinase (AMPK), inhibits growth in non-small cell lung cancer (NSCLC) cells [15]. Both inhibitors synergistically reduce the expression of SIRT1, increase acetylation and stability of p53, and, as a consequence, induce the expression of p53 downstream target proteins, such as p21, and growth arrest, and DNA damage-45 alpha (GADD45α), which subsequently promotes caspase3-dependent apoptosis (Figure 2D) [15].

#### 2.1.2. p53, SIRT1 and HDAC1 Expression Is Affected by miRNAs Influencing Cell Apoptosis

Except for SIRT1-mediated p53 deacetylation through protein–protein interactions, several miRNAs seem to affect SIRT1 and p53 expression and activity. In colon cancer, the miR-34a/SIRT1/p53 feedback loop is repressed by long-noncoding RNA (lncRNA) HNF1A-antisense 1 RNA1 (HNF1A-AS1). HNF1A-AS1 competitively binds miR-34a and increases SIRT1 expression, leading to the upregulation of the set of proteins involved in canonical Wnt signaling pathway and its activation, which ultimately promotes the metastatic progression of CRC (Figure 2E) [44,45]. In some cancer cells, miR-34a directly inhibits SIRT1 expression and parallelly induces the expression of p53, which leads to p53-mediated apoptosis and the suppression of cancer development (Figure 2F) [46]. In turn, a decrease in miR-204 expression in prostate cancer (PCa) cell lines and tissues upregulates SIRT1, enhancing the deacetylation of p53 [47]. miR-204 promotes the doxorubicin (DOX)-induced p53 acetylation through a decrease in SIRT1 expression. Acetylated p53 (p53-Ac) upregulates the expression of pro-apoptotic proteins Noxa and Puma and induces mitochondrial apoptosis (Figure 2F) [47]. The overexpression of miR-590-3p in breast cancer (BC) cells decreases the SIRT1 protein level, leading to an increased p53 level and its acetylation. It results in the upregulation of BAX and p21 expression, ultimately inducing apoptosis and suppressing cell survival (Figure 2F) [48]. Moreover, in BC cells, the miR34a, miR34c, and S-adenosyl-L-methionine (AdoMet) enhance p53 acetylation by decreasing SIRT1 and HDAC1 protein levels and potentiate apoptosis induced by AdoMet [49].

#### 2.1.3. SIRT3 Modulates p53 Tumor-Suppressive Functions

It is known that SIRT3 activity affects p53 protein status in cancer cells. However, several studies reported contradictory results that require further investigation. SIRT3 deacetylates and promotes lipid phosphatase activity of PTEN that reduces mouse double minute 2 homolog (MDM2) transcription and protects p53 from MDM2-mediated ubiquitin-proteasome degradation in BC and CRC cells [50]. In human hepatocellular carcinoma (HCC) tissues, the overexpression of SIRT3 increases the p53 protein level through the downregulation of MDM2 that reduces MDM2-dependent p53 degradation, indicating that SIRT3 acts as a tumor suppressor [51]. In contrast, some reports suggest that SIRT3 can decrease p53 protein expression by its direct deacetylation. It promotes p53 binding to MDM2 and increases p53 ubiquitination and degradation, contributing to malignancy in PTEN-deficient cancers (Figure 2G) [52]. Interestingly, it seems that p53 regulates the SIRT3 level affecting its post-transcriptional modification through proteasome-dependent mechanism [53]. A significantly higher SIRT3 protein level was observed in p53 wild-type (p53-WT) lung and CRC cancer cell lines in comparison to p53-deficient cell lines. In p53-depleted cells, increased level of S-phase kinase-associated protein 2 (SKP2) E3 ligase and enhanced turnover of SIRT3 were observed, suggesting that p53 regulates SIRT3 protein level through proteasome-pathway that involves SKP2 (Figure 2G) [53].

#### 2.1.4. SIRT6 and SIRT7 Directly Deacetylate p53 to Regulate p53-Mediated Apoptosis

SIRT6 deacetylates p53 at lysine 382, which may lead to ubiquitin-dependent p53 degradation, suggesting SIRT6 potential role in regulating stress resistance and apoptosis (Figure 2H) [54]. In HCC cells, SIRT7 interacts and deacetylates p53 at lysine 320 and lysine 373, by which it regulates doxorubicin (DOX)-induced apoptosis. The deacetylation of p53 decreases the Noxa transcription, thus blocking the 53-dependent apoptosis machinery in HHC in vitro model. In the mouse xenograft model, suppression of SIRT7 increases p53 activation induced by DOX, leading to apoptosis and inhibition of HCC growth [55]. Additionally, upregulation of SIRT7 expression is observed in HCC cell lines and patients’ tissues, correlating with shorter overall survival (Figure 2I) [55].

#### 2.1.5. HDAC1 and HDAC8 Deacetylate and Suppress p53 Activity

In addition to SIRTs, HDAC1 and HDAC8 influence the function of p53 by its deacetylation. In cutaneous T-cell lymphomas (CTLC) treatment with HDIs, such as valproic acid (VPA), trichostatin A (TSA), and PCI-34051, enhances p53 acetylation and mRNA levels of p53-regulated genes *Bcl-xl*, *NOXA,* and *PUMA* that are involved in p53-mediated apoptosis, emphasizing the importance of p53 as a respond target to HDIs [56]. Consequently, in glioma cells with AT-Rich Interaction Domain 4B (ARID4B) (oncoprotein involved in tumor progression) silencing, HDAC1 is upregulated, which leads to p53-Ac deacetylation suppressing cell apoptosis [57].

#### 2.1.6. HDAC1 and HDAC2 Control p53 Activity and Contribute to p53 Mutant Expression

In basal cell carcinoma (BCC) progenitors, the deletion of *HDAC1* and *HDAC2* significantly inhibits proliferation and enhances cell apoptosis, which is slightly restored after *PT53* or *P16* knockout (KO). A similar effect has been observed after non-selective HDI (romidepsin) treatment (normal epidermis and lesions cells) [58]. Considering that deletion of *PT53* partly salvages cell proliferation and apoptosis, the moderate effect of another pan-inhibitor, SAHA, towards tumor progression prevention, in a mice xenograft model with p53-depleted BCC cells is well-founded [59]. All these observations conclude that the efficiency in HDAC1 and HDAC2 inhibition is partly dependent on p53 and p16 status in BCC.

Since p53 mutations frequently appear in pancreatic cancer, they can be considered a potential anti-cancer therapeutic target. The last data showed that HDAC1 and HDAC2 contribute to p53 mutant (p53^R273H^) expression in vivo. In the murine pancreatic ductal adenocarcinoma (PDAC) model, the expression of p53 mRNA is reduced in HDAC1/2-deficient cells compared to normal cells, suggesting that HDACs upregulate p53 and maintain the mutant *TP53* transcription. Moreover, after SAHA treatment, which targets HDAC1 and HDAC2, p53^R273H^ expression is downregulated; therefore, SAHA-based therapy could have the potential for further solid tumors research [60]. In turn, in glioma cells, HDAC1 activity seems to be irrelevant in terms of p53-mutated cells. Unlike p53-WT cells, in p53-mutant cells, cell growth arrest and apoptosis are not observed after *HDAC1* silencing. Moreover, in p53-WT cells, *HDAC1*-KD affects cell phenotype changes in a p53-dependent manner [61]. These studies show the importance of HDAC1 in glioblastoma, indicating the high need to develop HDAC-specific isoform inhibitors.

Another research confirms the previous observations that HDAC2 could be a proper pharmacological target in tumors bearing p53 mutations [62]. HDAC2 is downregulated by RCY1, an E3 ligase involved in ubiquitin-mediated proteins degradation, in different cancer cells, including p53-WT, mutant, or p-53 depleted cells. Moreover, HDAC2 expression is enhanced by RCHY1 knockdown, and an inverse correlation between RCHY1 and HDAC2 levels was found in patients’ samples [62]. In turn, silencing of *HDAC2* reduces the ATM/p53-mediated cell death in osteosarcoma cells after DOX treatment, which suggests that HDAC2 takes part in DNA early damage response and could be a coactivator for p53. In detail, DOX-induced cell apoptosis in p53-WT cells is attended by significant p53 and H2A histone family member X (H2AX) accumulation. Silencing of *HDAC2* meaningfully deteriorates sensitivity to DOX and reduces p53-mediated DNA damage responses through downregulation of ATM and p53 (at Ser-15) phosphorylation. Taking into account that ATM-mediated H2AX phosphorylation occurs during early DNA damage response, and p53 is a substrate for ATM-mediated propagation of DNA damage signaling, HDAC2 may be involved in ATM activation. Interestingly, silencing *HDAC2* in p53-null lung cancer cells shows a weak effect of DOX-mediated phosphorylation of ATM, proving that HDAC2 influence on ATM depends on p53 [63]. The HDAC2 impact on DNA-damaging agents resistance has also been established for colorectal adenocarcinoma CRC cell lines with different statuses of *TP53* (*TP53*-mutated, *TP53*-WT, and *TP53*-deficient cells) by determining the pharmacological effect between them and SAHA or VPA. In untreated p53-mutated cells, the HDAC2 expression is lower in comparison to p53-WT cells. Upregulated HDAC2 expression is associated with drug resistance, and HDAC2 depletion sensitizes multidrug-resistant cells (HT-29) to chemotherapeutic agents (5-FU or oxaliplatin) commonly used in CRC treatment. Combined treatment with SAHA and 5-FU or oxaliplatin downregulates HDAC2 expression, inducing cell apoptosis. The synergetic effect of combined treatment has been validated in vivo, where xenograft tumor growth is reduced by half after drugs treatment. These results speculate that CRC outcomes after combined treatment with HDIs and DNA-damage agents depend more on HDAC2 expression than p53 mutation status [64]. The effect of VPA has been also investigated against HCC cell lines, showing that VPA downregulates *HDAC1/2/3* and upregulated *P21* and *PT53* gene expression simultaneously, which resulted in cell apoptosis [65].

### 2.2. SIRT1, SIRT2, and HDAC1 Deacetylate p73 and Suppress Its Activity

The p73 protein, a homolog of p53, induces cell apoptosis and cell cycle arrest [66]. p73 is rarely mutated in tumors [67], and in vivo research indicates that p73-deficiency does not increase the tumor incidence. Nonetheless, a few studies reported reduced expression or depletion of p73 in specific tumors, suggesting that p73 acts as a tumor suppressor [68].

SIRTs participate in this regulation through their activity against p73 and transcription factor E2F1 (E2F1) [69]. They are able to regulate tumor suppressors directly: as a result of deacetylation of p73 by SIRT1, or indirectly as a result of deacetylation of E2F1, and thus the inhibition of p73 activation [69]. Thereby, the modulation of the p73 network may help overcome chemoresistance in human cancers, as the inactivation of the *TP73* gene is associated with an increased chemoresistance [69,70]. SIRT1 binding to p73 suppresses its transcriptional activity and partly inhibits p73-dependent apoptosis in HeLa cell lines [70]. Deacetylation of p73-Ac by SIRT1 inhibits p73-dependent BAX transcription suggesting that abnormal expression of SIRT1 promotes tumorigenesis (Figure 2J) [70]. SIRT2 also deacetylates p73 at C-terminal lysines, which suppresses its transcriptional activity and increases tumorigenicity and the proliferation of glioblastoma cells (Figure 2J) [71]. The overexpression of p73 protein and suppression of SIRT2 activity with one of the specific inhibitors (AGK2 or AK7) results in the induction of apoptosis [71]. Except for SIRTs, HDAC1 is also involved in p73 deacetylation. A simultaneous ectopic expression of p73 and silencing of the *HDAC1* gene in metastatic melanoma cells results in enhancement of apoptosis and autophagy (Figure 2J) [72]. The biological effects of the p53-Ac and p73-Ac deacetylation catalyzed by SIRTs and HDACs are presented in Figure 2 [18,19,20,21,22,23,24,25,26,27,28,29,30,31,32,33,34,35,36,37,38,39].

### 2.3. FOXO Deacetylation by HDACs Gives Different Biological Effects

The family of forkhead (FOXO) proteins is a set of transcription factors that possess a highly conserved DNA-binding domain called the “forkhead box” (FOX). FOXO family regulates a wide range of cellular processes such as stress resistance, apoptosis, and cell cycle arrest. Among FOXO proteins, FOXO3a is a tumor suppressor regulating cell survival or death in response to chemotherapy and metabolic stress [73,74].

#### 2.3.1. FOXO3a Is Deacetylated by SIRT1 and SIRT7 That Regulates Apoptosis

**Figure 3:**
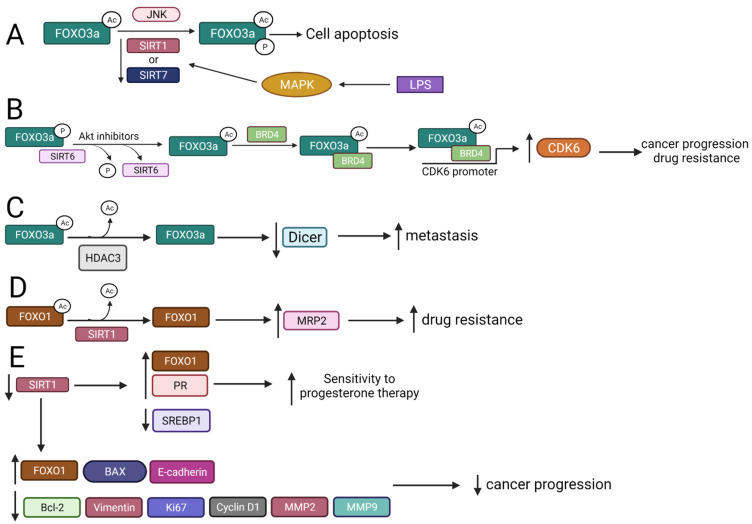
The interplay between SIRTs, HDACs, and FOXOs and the biological results of these interactions. ↑—upregulation, ↓—downregulation (**A**) Downregulation of SIRT1 and SIRT7 by LPS treatment increases FOXO3a acetylation and phosphorylation, which induces apoptosis. (**B**) Akt inhibitors interrupt phosphorylated FOXO3a–SIRT6 interaction, leading to FOXO3a acetylation and BRD4 binding, promoting upregulation of CDK6, cancer progression, and drug resistance. (**C**) FOXO3a is deacetylated by HDAC3, which leads to Dicer downregulation and enhances metastasis. (**D**) SIRT1 deacetylates FOXO1, leading to MRP2 upregulation and enhanced drug resistance. (**E**) Downregulation of SIRT1 upregulates FOXO1, leading to increased sensitivity to progesterone therapy, and modulates expression of genes involved in the regulation of cancer growth, angiogenesis, cell survival, EMT, cell proliferation, and cancer invasion.LPS—lipopolysaccharide; BRD4—bromodomain-containing protein 4; CDK6—cyclin dependent kinase 6; MRP2—multidrug resistance protein 2; EMT—epithelial-mesenchymal transition.

Both SIRT1 and SIRT7 can deacetylate FOXO3a, which prevents its phosphorylation at serine 574 (S574) and blocks lipopolysaccharide (LPS)-induced apoptosis in leukemic monocytes [74]. The formation of phosphorylated FOXO3a (FOXO3a-P) is regulated by the state of its acetylation, wherein acetylated FOXO3a favorably interacts with c-Jun N-terminal kinase (JNK1), resulting in FOXO3a phosphorylation. Deacetylated FOXO3a is maintained mainly due to SIRT1 and SIRT7 deacetylase activity. SIRT1 and SIRT7 stability is reduced by LPS-induced signaling that involves a mitogen-activated protein kinase (MAPK) pathway, leading to an increase in FOXO3a-P level and induction of cell apoptosis (Figure 3A) [74].

FOXO3a can increase *Bim* expression and stimulate lung cancer cells’ apoptosis, which is partly mediated by early growth response protein 1 (EGR1), which binds to the *Bim* promoter region. SIRT1 stimulates this pro-apoptotic effect through deacetylation of FOXO3a-Ac, which induces EGR1 binding to the *Bim* promoter and *Bim* expression [75]. Conversely, in glioma cells, a reduction in SIRT1 expression leads to increased acetylation of FOXO3a and a higher expression of Bim and PUMA, resulting in decreased proliferation, viability, and an induction of apoptosis [76].

#### 2.3.2. SIRT6 Interacts with FOXO3a to Regulate Cancer Progression, Drug Resistance, and Apoptosis

The role of the SIRT6 and FOXO3a/bromodomain-containing protein 4 (BRD4) axis is underlined in the progression and drug resistance of luminal BC [77]. BRD4 co-creates transcriptional machinery controlling the expression of genes critical for tumor progression. Furthermore, SIRT6/FOXO3a/BRD4/ cyclin-dependent kinase 6 (CDK6) axis participates in the development of resistance to Akt inhibitors. Akt inhibitors are able to induce dephosphorylation of FOXO3a and disrupt its interaction with SIRT6, leading to acetylation of FOXO3a (FOXO3a-Ac). FOXO3a-Ac recruits the BRD4/RNAPII complex to the *CDK6* gene promoter region, inducing its transcription. The inhibition of either CDK6 activity or BRD4/FOXO3a association overcomes the resistance of luminal BC cells to Akt inhibitors in vitro and in vivo (Figure 3B) [77].

In human CRC cell lines, FOXO3a positively regulates SIRT6 expression, leading to BAX-induced apoptosis [78]. The FOXO3a activity is increased by inactivation of Akt, augmenting its affinity and binding to the SIRT6 promoter that in turn enhances SIRT6 expression. SIRT6 induces apoptosis probably through deacetylation of lysine residues on histone 3 (H3K9) in the survivin gene promoter that leads to inhibition of anti-apoptotic protein survivin expression. The SIRT6 knockdown (SIRT6-KD) abrogates apoptotic responses and grants resistance against PI3K inhibitor (BKM120), indicating that inactivation of Akt and induction of SIRT6 may become a novel combined CRC therapy [78].

#### 2.3.3. HDAC3 Interplays with FOXO3 Increasing Metastasis

In the BC LM2 cell line, the geminin, a DNA replication inhibitor, selectively tethers FOXO3a to HDAC3, which results in the deacetylation and inactivation of FOXO3a transcriptional activity leading to downregulation of the FOXO3a target Dicer, which is an RNase that suppresses metastasis [79]. The silencing of HDAC3 or depletion of geminin decreases the migration/invasion potential of LM2 cells as well as their metastatic capacity in vivo. These findings indicate the importance of the FOXO3a/Dicer axis as a downstream effector of geminin/HDAC3-dependent BC metastasis (Figure 3C) [79].

#### 2.3.4. SIRT1 Mediates FOXO1 Deacetylation

The regulation of FOXO1 activity by SIRT1 yields opposite biological effects in different cancer cells, emphasizing an individual role of HDACs in tumorigenesis and the response to treatment. In BC cells, FOXO1 nuclear localization is regulated by SIRT1 deacetylase activity, where FOXO1 upregulates multidrug resistance protein 2 (*MRP2*) gene expression. The overexpression of both SIRT1 and FOXO1 enhances transcription of the *MRP2* gene, while the inhibition of SIRT1 decreased both the MRP2 expression and FOXO1 nuclear levels that increases the cytotoxic effect of chemotherapeutic agents, such as DOX and paclitaxel (PAX) (Figure 3D) [80]. Contrary, in progestin-resistant endometrial cancer (EC) cells, SIRT1 knockout (SIRT1-KO) results in the upregulation of progesterone receptor (PR) and FOXO1, as well as the downregulation of sterol regulatory element-binding protein-1 (SREBP-1) that increases sensitivity to progestin therapy. Therefore, targeting the SIRT1/FOXO1/SREBP-1 pathway that regulates PR may help overcome progestin resistance in EC cells (Figure 3E) [81].

In gastric cancer (GC), SIRT1 silencing or inhibiting its activity by EX527 enhances expression of FOXO1, pro-apoptotic BAX, and E-cadherin, whereas the expression of Ki67, Cyclin D1, anti-apoptotic Bcl-2, Vimentin, MMP-2 and MMP-9 are downregulated, suggesting SIRT1 involvement in GC progression (Figure 3E) [82]. In turn, treatment of HCC cells (HepG2 and Huh7) with cambinol (SIRT 1/2 Inhibitor) or EX-527 (a selective SIRT1 inhibitor) increases the levels of acetylated FOXO1 and p53, reduces cellular viability and migration [83].

#### 2.3.5. FOXOM1 Is Acetylated by CBP/p300

Among HDACs, HATs are also involved in non-histone protein modification. CBP/p300 acetylates FOXM1 at several lysine residues (63, 422, 440, 603, and 614), which is essential for transcriptional activation of its target genes. The FOXM1 acetylation increases the activity of this protein by increasing its stability, DNA binding affinity, and sensitivity to phosphorylation. Additionally, the acetylation of FOXM1 promotes the proliferation of cervical cancer (HeLa) cells and tumor growth in vivo. On the other hand, SIRT1 acts as a negative cofactor for FOXM1 as it can deacetylate FOXM1, which decreases its stability and transcriptional activity. Therefore, activating SIRT1 to deacetylate FOXM1 may become an efficient strategy for treating cancers with overexpressed FOXM1 [84].

## 3. Oncoproteins

### 3.1. HDACs Deacetylate NF-ĸB Family Member p65 Modulating Its Tumor-Suppressive Functions

The nuclear factor kappa-light-chain-enhancer of activated B cells (NF-ĸB) family is known as an important regulator of gene expression involved in inflammatory processes, cell proliferation, and apoptosis [85]. The NF-ĸB network consists of five protein monomers (p65, p52, p50, RelB, cRel) that can form homodimers or heterodimers that bind to DNA [86]. NF-ĸB function is affected by HAT-mediated acetylation and HDAC-mediated deacetylation of p65 [17,87,88,89].

#### 3.1.1. p65 Activity Is Modulated by SIRT1 and SIRT2 That Inhibits Cell Cancer Growth

**Figure 4:**
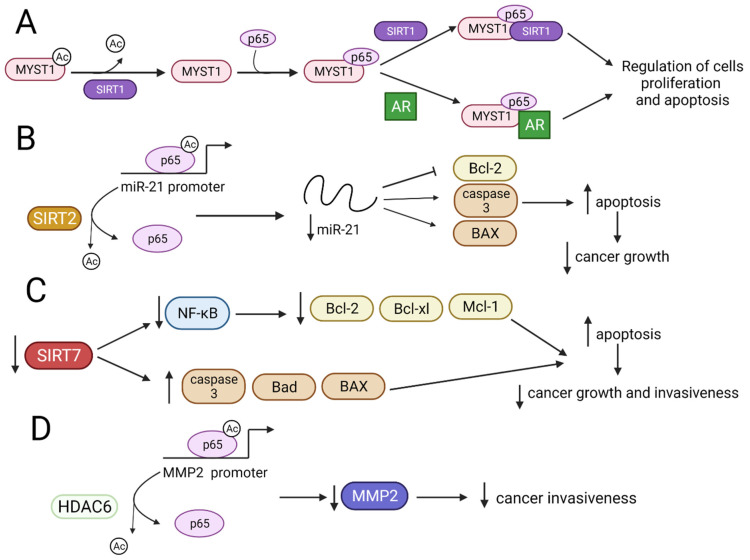
The influence of HDACs and SIRTs on NF-κB and biological results of these modifications. ↑—upregulation, ↓—downregulation, ¬ inhibition, (**A**) SIRT1 deacetylates MYST1-Ac, which interacts with p65. The MYST1–p65 complex binds SIRT1 or AR, thus modulating the intensity of cell proliferation and apoptosis. (**B**) SIRT2 deacetylates p65-Ac, leading to downregulation of miR-21 expression and inhibition of cancer growth. (**C**) Downregulation of SIRT7 leads to a decrease in N.F.- κB, Bcl-xl, Bcl-2, and Mcl-1 expressions, and the upregulation of caspase-3 and Bad and BAX proteins induces apoptosis and inhibits the growth and invasiveness of cancer cells. (**D**) HDAC6 deacetylates p65-Ac, preventing p65 from binding to the MMP2 promoter region and ultimately decreasing cancer invasiveness.AR—androgen receptor; N.F.- κB—nuclear factor kappa-light-chain-enhancer of activated B cells; Mcl-1—myeloid cell leukaemia-1; MMP2- matrix metalloproteinase-2.

MYST1, a member of the MYST family containing a HAT domain, acts as a coactivator of NF-κB in PCa cells [17]. SIRT1 interacts with MYST1 and downregulates its autoacetylation forming the MYST1–p65–SIRT1 complex. Simultaneously, MYST1 interacts with p65 and androgen receptor (AR) to regulate tumor behavior. Due to mutually exclusive MYST1 interactions, both complexes act opposite to each other (MYST1–p65–SIRT1 act as a repressor complex, while MYST1–p65–AR as an activator complex), controlling the acetylation of lysine 16 on histone H4 (H4K16Ac) involved in the regulation of cancer progression. MYST1–p65–SIRT1 complex represses apoptotic pathways enhancing cell proliferation and metastasis, while MYST1–p65–AR complex upregulates p21 protein expression leading to G_2_M phase arrest during cell cycle progression, resulting in the inhibition of PCa growth (Figure 4A) [17]. In human glioma tumors and cell lines, SIRT2 deacetylates p65 at lysine 310 and inhibits miR-21 transcription through blocking p65 binding to the miR-21 promoter, suppressing the growth of glioma cells (Figure 4B) [87].

#### 3.1.2. Downregulation of SIRT7 Gene Decreases Expression of NF-κB and Inhibits the Growth and Invasiveness of Cancer Cells

The downregulation of *SIRT7* decreases the expression of NF-κB and its target proteins, including anti-apoptotic Bcl-xl, Bcl-2, and Mcl-1, and increases pro-apoptotic proteins, such as caspase-3, Bad, and BAX, inhibiting the growth and invasiveness of endometrial cancer cells (Figure 4C) [88]. In contrast to SIRT7, the ectopic expression of nuclear HDAC6 in NSCLC cells inhibits cancer invasiveness by the deacetylation of p65, which, in turn, decreases its binding to the matrix metalloproteinase-2 (MMP2) promoter and reduces *MMP2* expression (Figure 4D) [89]. The mechanisms involved in NF-κB acetylation and MYST1 deacetylation by SIRT1, SIRT2, SIRT7 and HDAC6 are presented in Figure 4 [17,87,88,89].

#### 3.1.3. HDIs Regulate Expression of NF-κB Partly through Inhibition of HDACs Activity

In liver cancer cells, the inhibition of class I HDACs by a natural compound called hydroxygenkwanin (HGK) increases p65 acetylation at K310, promoting its activation and ultimately upregulating the expression of its downstream tumor suppressor genes (such as *DR5*). Since the acetylation of p65 at K310 could be considered an indicator of p65 anti-cancer activity, the marked increase in the p65-Ac level after HGK treatment indicates the anti-cancer potential of this compound. [90].In turn, in myeloma cells, the use of CUDC-907 compound, a dual inhibitor for HDACs 1/2/3/10 and PI3K, leads to the reduction in p65 expression in a CUDC-907-dependent manner. As the upregulation of NF-κB activity corresponds with chemoresistance, the decrease in NF-κB expression could be a valuable target in anti-myeloma treatment [91]. Additionally, the therapeutical efficiency for CUDC-907 has been proven for human T-cell leukemia virus type 1 (HTLV-1)-driven adult T-cell leukemia (ATL). CUDC-907 inhibits the expression of multiple pro-survival proteins and also inhibits NF-κB expression [92]. The downregulation of NF-κB is also observed when another HDI is used. The use of romidepsin (HDAC1/2 inhibitor) causes a significant enhancement of CYLD, a negative regulator for NF-κB, in an HCC mice model, which partly explains the NF-κB downregulation. In conclusion, romidepsin suppresses the early stage of HCC. The suggested mechanism could be associated with the tumor-suppression activity of romidepsin through the deregulation of critical cancer-related proteins, including NF-κB [93]. In line with these observations, the subsequent finding indicates that the activation of the NF-κB signaling pathway is observed in Kaposi’s sarcoma (KS), an endothelial spindle-shaped cell tumor induced by KS-associated herpesvirus (KSHV), when HDAC1 is downregulated. In detail, an oncogenic protein called KSHV-encoded viral FLICE-inhibitory protein (vFLIP) leads to the degradation of the histone deacetylase complex subunit (SAP18), a component of the histone deacetylase complex, which includes, among others, HDAC1 and HDAC2. Meanwhile, transcription factor Nanog, known as an HDAC1 promoter, is inhibited by vFLIP, which resulted in HDAC1 downregulation. Ultimately, the downregulation of the SAP18/HDAC1 complex increased p65 acetylation, activating the NF-κB signaling pathway and thus inducing cancer progression and angiogenesis [94].

### 3.2. Signal Transducers and Activators of Transcription (STATs)

Signal transducers and activators of transcription (STATs) constitute a family of proteins (STAT1-4, STAT5A, STAT5B, and STAT6) responsible for regulating gene expression. Activation of STAT proteins occurs due to their phosphorylation by receptor-associated Janus kinases followed by protein dimerization, transportation of formed dimers to the nucleus, and their binding to DNA within the promoter regions. This, in turn, results in the expression of multiple genes. However, STATs activation can also inhibit specific genes, such as those encoding matrix metalloproteinases and genes involved in cell cycle progression. Therefore, they play the role of a linker connecting multiple signal transduction pathways, and they are essential in many biological processes such as cellular growth, differentiation, apoptosis, and immunity. Increased STAT3 activity is observed in more than 50% of malignancies, including breast, ovarian, lung, prostate cancer, leukemia, and lymphoma [95].

#### 3.2.1. HDAC1 and HDAC4 Inhibit STAT3 Activity and Interfere with Its Stability

**Figure 5:**
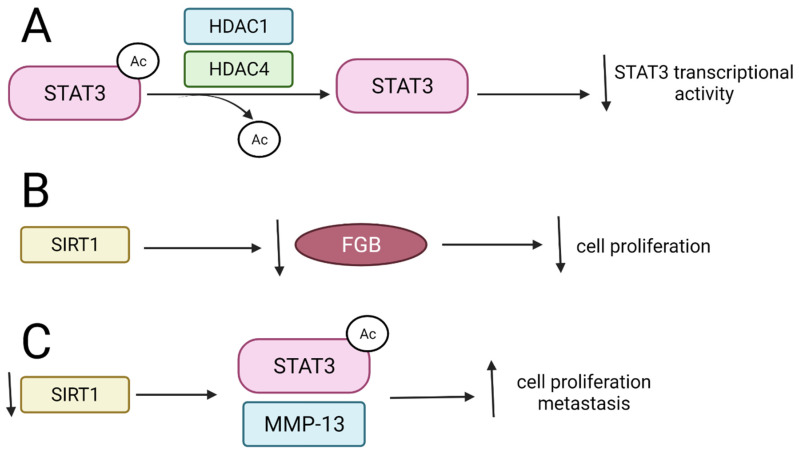
The influence of SIRTs and HDACs on STAT deacetylation and biological results of these modifications. ↑—upregulation, ↓—downregulation. (**A**) HDAC1 and HDAC4 deacetylate STAT3-Ac leading to decrease in STAT3 transcriptional activity; (**B**) SIRT1 represses FGB leading to inhibition of cell proliferation; (**C**) Downregulation of SIRT1 results in upregulation of STAT3-Ac and MMP-13 what enhances cell proliferation.STAT—signal transducers and activators of transcription; FGB—fibrinogen beta chain; MMP13-matrix metalloproteinase 13.

STAT3 is acetylated by histone acetyltransferase p300 at lysine 49 and 87. HDAC1, on the other hand, is involved in the deacetylation process, resulting in the inhibition of STAT3 transcriptional activity in human prostate cancer (PC3) cell lines [96]. Furthermore, HDACs 1 and 4 are responsible for the deacetylation of STAT3 to either terminate STAT3 transcriptional activity or maintain the deacetylated form of STAT3 (Figure 5A) [97].

#### 3.2.2. SIRT1 and Its Activators Affect STAT3 Transcriptional Function

SIRT1 deacetylates STAT3, which promotes the degradation of STAT3 and leads to the suppression of tumorigenesis in renal cell carcinoma (RCC) [98]. SIRT1 inhibits RCC proliferation by deacetylating and thus destabilizing STAT3, which in turn leads to the inhibition of *FGB* gene expression. The *FGB* gene encodes the fibrinogen Bβ chains, and it constitutes a target gene for STAT3. Overexpression of FGB protein resulting from an increased STAT3 expression is observed in patients with RCC, and it is associated with tumor progression and poor prognosis [98] (Figure 5B). Depletion of SIRT1 increases STAT3 acetylation and phosphorylation as well as upregulates matrix metalloproteinase 13 (MMP-13) protein in gastric cancer (GC) both in vivo and in vitro, which, together with other metalloproteinases such as MMP-2 and MMP-9, play an important role in cancer cell invasion via the degradation of the extracellular matrix. The activation of STAT3/MMP13 signaling after SIRT1 depletion suggests that SIRT1 may work as a tumor suppressor (Figure 5C) [19].

Additionally, using SIRT1 activators, SRT501 and SRT2183, results in the growth, inhibition, and induction of apoptosis in malignant lymphoid cells through the upregulation of growth arrest DNA-damage-inducible protein GADD45 gamma (GADD45G). This, in turn, is due to the inhibition of binding of NF-κB/STAT3 complex to the GADD45G promoter [99]. Nevertheless, the mechanism of SIRT1 activation using the compounds mentioned above remains a matter of dispute. The most likely and accepted mechanism of action of these activators is the allosteric mechanism consisting of conformational changes within the N-terminal domain of SIRT1. This leads to better binding of SIRT1 to its substrates [100]. In line with these findings, SIRT1 activators seem to be a promising tool in anticancer treatment. In research focused on gastric cancer (GC), the use of SIRT1 activator resveratrol (RSV) resulted in a significant reduction in *STAT3* and *c-myc* gene expression as well as the expression of phosphorylated (STAT3-P) and acetylated (STAT3-Ac) forms of STAT3. RSV significantly decreases cell viability and facilitates senescence in GC cell lines as compared to the normal gastric cell line [101]. Correspondingly, new SIRT1 activators: SRT2183 and SRT501 induce the deacetylation of STAT3, apoptosis, and growth arrest in malignant lymphoid cells with the constitutively activated STAT3 signaling pathway [99].

Since STAT3 regulates the expression of proinflammatory genes, the inhibition of its activity against cytokines secreted by Th17 cells should be emphasized. However, the role of Th17 cells in neoplasms remains controversial and elusive, as they exhibit oncogenic properties in certain types of malignancies yet suppress the development of other tumors. Th-17 cells secrete, among others, IL-17A and IL-17F proinflammatory cytokines, and STAT3 can directly regulate their expression by binding to their promoter regions. The treatment of patients affected by metastatic colon cancer with metformin (SIRT1 agonist) revealed decreased acetylation of STAT3, impeded Th17 cell differentiation, and reduced secretion of IL-17A cytokine by Th-17 cells. Additionally, in vivo studies showed that the use of metformin resulted in reduced tumor growth in a SIRT1-dependent manner [102]. These findings indicate that SIRT1 may function as a tumor suppressor in the tumorigenesis of different cancers, and SIRT1 activators constitute potential therapeutic tools that may be considered for cancer treatment in the future.

### 3.3. Myc Family

**Figure 6:**
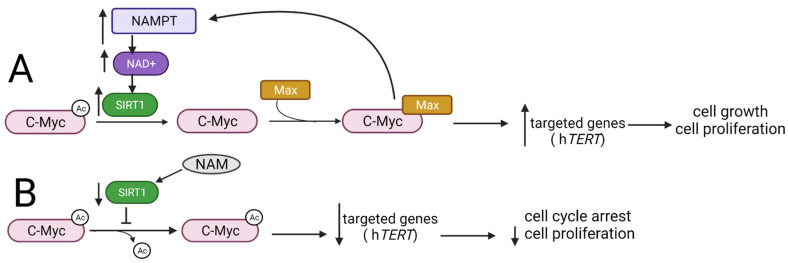
The interplay between SIRT1 and Myc proteins. (**A**) SIRT1 deacetylates C-Myc and thus enables the formation of C-Myc/Max heterodimers, resulting in C-Myc transactivation, followed by an increased expression of its target genes and thus stimulation of cell growth and proliferation. Simultaneously, C-Myc stimulates the activity of SIRT1 by inducing the expression of the *NAMPT* gene encoding the enzyme responsible for the production of the SIRT1 cofactor NAD^+^. (**B**) The use of the NAM leads to the inhibition of SIRT1 activity and thus inhibits C-Myc protein deacetylation, decreases the expression of target genes, and results in cell cycle arrest. NAMPT—nicotinamide-phosphoribosyltransferase; NAD^+^—nicotinamide adenine dinucleotide; hTERT—human telomerase reverse transcriptase; NAM—nicotinamide.

Another group of oncoproteins that are post-translationally regulated via acetylation and deacetylation consists of three members in the Myc family that are encoded by *c-myc*, *l-myc,* and *n-myc* genes [103]. Myc proteins regulate genes involved in cell proliferation, differentiation, intercellular communication, and cell cycle control. Dysregulation of Myc gene expression or protein stabilization is found in many types of cancers [104]. c-Myc binds to the promoter of SIRT1 and increases SIRT1 expression. SIRT1 interacts and deacetylases c-Myc, decreasing its stability, which suggests that SIRT1 plays a role in tumor suppression [105]. On the other hand, the binding of SIRT1 to the carboxyterminal domain of c-Myc and its deacetylation by SIRT1 leads to the increased formation of c-Myc/myc-associated factor X (Max) heterodimers, which, in turn, facilitates the transactivation of c-Myc (Figure 6A) [106]. The formation of the C-Myc/Max heterodimers is necessary for the recognition of the target gene promoter by the C-Myc protein, and thus, it constitutes a necessary condition for the proper performance of its function as a transcription factor. One of the C-Myc target genes is the *hTERT* gene encoding the human telomerase reverse transcriptase, responsible for the synthesis of the telomerase catalytic subunit. Interestingly, TERT enables the binding of C-Myc to the target gene promoter and plays a crucial role in its stabilization [107]. Furthermore, the active C-Myc/Max complex induces expression of the *NAMPT* gene encoding nicotinamide-phosphoribosyltransferase. This, in turn, leads to an increase in NAD^+^, which is a cofactor for SIRT1, resulting in an increase in the level of the SIRT1 protein [108].

The anticancer effect of the SIRT1 inhibitor comes from the decreased expression of c-Myc target genes, which leads to suppressed proliferation and induction of cell cycle arrest at the G1/S phase in leukemic cells [106]. Similarly, the use of SIRT2 specific inhibitor: TM (thiomyristoyl lysine compound) stimulates c-Myc ubiquitination and its degradation in various cancer cell lines depending on the sensitivity of cells to TM. Interestingly, a negligible effect of TM action is observed both in non-tumor cells and in tumor-free mice, indicating a greater dependence of cancer cells on SIRT2, which may indicate SIRT2 as a potential therapeutic target [109]. Mao and colleagues revealed that the use of nicotinamide (NAM), the precursor for the synthesis of NAD^+^, leads to the inhibition of SIRT1 activity and thus decrease in the C-Myc protein expression (Figure 6B) [106].

## 4. Concluding Remarks and Future Perspectives

HDACs play a prominent role in regulating gene expression not only through their ability to deacetylate histones but also because of deacetylation of other nuclear (or cytosolic) proteins, including both tumor suppressors and oncoproteins, which, in turn, influences their stability and function. Thereby, epigenetic alterations of non-histone proteins, including TFs are regarded as promising tools for anti-cancer therapy. Recently, targeted therapy, as well as immunotherapy, improved patients outcomes and their survival in various cancer types [110].

The inhibition of particular deacetylases by their specific inhibitors seems to have clinical importance in the anti-cancer treatment [111]. A considerable number of studies have shown a controversial role of HDACs in tumorigenesis, either in promoting cancer cell survival or causing cell death among different types of cancers, as described in this review. Moreover, some research [53,112,113,114] indicates that HDACs can not only modulate the function of non-histone proteins, but reversibly, they are regulated by these proteins. The inhibition of SIRT1 to restore p53 activity [115] or to reverse chemoresistance of lung cancer cells is only one of such examples. Although, until now, none of SIRT1 chemical inhibitors has been approved for patients’ treatment, very recently, nonsteroidal anti-inflammatory drugs (NSAIDs) have been shown to inhibit the SIRT1 deacetylase activity, augmenting acetylation and activity of the p53, suggesting a novel strategy for breast cancer treatment [116]. Given that both types ofTFs—tumor suppressors and oncoproteins—undergo post-translational modifications, HDAC activators also become a promising tool in anti-cancer therapy. In some types of cancer, higher expression of HDACs leads to the inactivation or/and degradation of oncoproteins [19,98]. Modifying the acetylation/deacetylation status of signaling molecules and transcription factors by classical HDACs (HDAC1/2/3/6) opens the possibility for patients’ treatment with HDIs already approved for clinical use, including VPA, SAHA, or pabinostat [117]. However, despite several ongoing clinical trials where HDIs are included in the treatment strategy, there is little or no information about the acetylation status of non-histone proteins in cancer patients during medication. The single report demonstrated that the combination of pan-HDAC inhibitors with niacinamide produced remissions in a spontaneous aggressive B-cell lymphoma mouse model and correlated with the acetylation of both Bcl6 and p53 [118]. This finding opens a possibility to utilize such kind of information for clinical purposes. Specifically, modified (acetylated or deacetylated) non-histone proteins could serve as potential cancer markers, both for diagnosis or/and monitoring of treatment efficacy, especially when HDIs are included in treatment schemes. Additionally, this type of molecular profiling could allow predicting the functionality of crucial tumor suppressors and oncogenes apart from their mutation status only, providing clues for therapy optimization. Although the HDACs’ activity against histone proteins is well-described in literature, there are missing points in the effect of HDACs’ activity on histone and non-histone proteins simultaneously. The HDIs have different specificity towards HDACs, and therefore, they change the acetylation of different regulatory proteins and influence many cellular functions [119]. Additionally, biological outcomes of a treatment with HDIs depend not only on the type of cancer cells but the presence of mutations in tumor suppressors or/and oncogenes [120]. A better understanding of biological effects induced by post-translational modifications of histone and non-histone proteins will help find the best HDIs for targeted therapy in patients with different tumors and predict the response to such treatment.

The role of acetylation/deacetylation of non-histone proteins is still not fully understood and needs further investigation to develop new drugs for targeted anti-cancer therapy.
